# Clinics and Practice: Consolidating Best Practices in Periodontal Management

**DOI:** 10.3390/clinpract13030061

**Published:** 2023-06-02

**Authors:** Francesco D’Ambrosio

**Affiliations:** Department of Medicine, Surgery and Dentistry, University of Salerno, 84084 Salerno, Italy; fradambrosio@unisa.it or francesco87fd@libero.it

Periodontitis is a multifactorial inflammatory disease [1] that results in periodontal attachment and bone loss. As a disorder, it occurs as a secondary development to the immune–inflammatory reaction established within the periodontal tissues in response to microbial insult [2].

The strategies for the primary prevention of periodontitis are mainly based on biofilm control and the reduction of identified risk factors. Indeed, in addition to genetics, modifiable factors, including unhealthy behaviors, such as alcohol consumption and smoking [3], and compromising systemic conditions, such as obesity, as well as disorders, including diabetes mellitus, metabolic syndrome, and osteoporosis [2,4,5], influence the individual response to periodontal infection.

The identification and control of such general risk factors should be considered crucial for periodontal health management, from both preventive and therapeutic points of view [6]. This is especially the case when it is considered that smoking and diabetes have also been recognized as periodontitis-grade modifiers [2,7] with the capacity to affect the rate of periodontitis progression.

Coherently, it has long been known that various systemic conditions and disorders can affect periodontal support tissues [8,9,10]. Specifically, periodontitis may comprise a manifestation of several systemic diseases, including rare conditions that lead to early-onset and severe forms of periodontitis. Additionally, several common systemic conditions and disorders may impact periodontitis occurrence, severity, and progression [7,11,12]. The relationship between periodontal and general health status is mainly attributed to both dysbiotic phenomena and inflammatory status, factors which may be responsible for the multilayered pathogenic links between periodontitis and degenerative, inflammatory, and neoplastic diseases at distant sites [13,14]. Indeed, the suspected periodontal pathogens within the gingival biofilm are able to act indirectly through endotoxin production. Alternatively, they are able to enter the systemic circulation, reach distant organs and act directly through their virulence factors [9]. In addition, pro-inflammatory cytokines released in the inflamed periodontal tissues during periodontitis have the capacity to influence the systemic inflammatory state. Analogously, systemic inflammatory cytokines can reach the periodontal tissues and, on a secondary level, influence periodontal health status and response to periodontal treatment [8,9].

The treatment of periodontitis aims to prevent further disease progression, restore lost tissue wherever possible, and maintain healthy periodontal conditions [15]. Given the chronic nature of the disease, achieving and maintaining a healthy periodontal condition requires lifelong patient education, motivation, and guidance in appropriate periodontal self-care, as well as a combination of therapeutic approaches based on periodontitis staging and grading [15].

Therapeutic approaches provide mechanical biofilm and calculus removal via supragingival and subgingival instrumentation [16,17]; these are supported, when necessary, by chemical control using antiseptics [18,19,20], systemic and local antimicrobial adjuncts [21,22], and surgical treatment in advanced disease stages [23,24]. Specifically, periodontal surgical reconstructive/regenerative (bone grafting, enamel matrix derivative, guided tissue regeneration, and blood-derived growth factor therapy) procedures have shown greater CAL gains in intrabony defects compared with open-flap debridement [25], especially in combined approaches and with papillary preservation flaps [23,26,27], even in the long term (5–20 years) [24].

Moreover, several conventional and novel anti-inflammatory drugs, antioxidants, and other biologic agents [28], including probiotics, have been proposed for use in control periodontal inflammation through host modulation [29,30]. Drugs administered in systemic diseases, such as statins, whether used alone or in combination with nonsurgical periodontal treatment, are also being investigated for their potential beneficial effects on periodontal health [28,31].

Furthermore, the capacity to control systemic conditions or disorders that potentially affect tooth-supporting structures may positively influence periodontal outcomes after mechanical treatment, as previously demonstrated for glycemic control in periodontal diabetic patients [7]. Similarly, since periodontal health is part of general health [13], treating periodontitis has been proven to ameliorate systemic health and reduce cardiometabolic risk, systemic inflammation, and preterm birth [32].

In parallel with advances in understanding the mechanisms underlying the etiopathogenesis of periodontitis, more effective and efficient approaches are being developed for managing periodontitis. These breakthroughs may be particularly relevant for recurrent and refractory cases, and may even enable practitioners to reduce or hopefully halt periodontal tissue destruction to avoid severe jaw atrophy and the resulting complex rehabilitation process [33,34,35]. The complex relationships between general and periodontal health emphasizes the role played by therapeutic approaches in the active and maintenance phases of periodontal treatment tailored to a patient’s systemic conditions [36,37].

## Data Availability

Data are available on PubMed/MEDLINE, Scopus and Web of Science databases.
